# MRTF-A-NF-κB/p65 axis-mediated PDL1 transcription and expression contributes to immune evasion of non-small-cell lung cancer via TGF-β

**DOI:** 10.1038/s12276-021-00670-3

**Published:** 2021-09-21

**Authors:** Fu Du, Xin Qi, Aotong Zhang, Fanfan Sui, Xuemin Wang, Christopher G. Proud, Cunzhi Lin, Xinglong Fan, Jing Li

**Affiliations:** 1 Key Laboratory of Marine Drugs, Chinese Ministry of Education, School of Medicine and Pharmacy, Ocean University of China, Qingdao, 266003 People’s Republic of China; 2 Laboratory for Marine Drugs and Bioproducts of Qingdao National Laboratory for Marine Science and Technology, Qingdao, 266237 People’s Republic of China; 3 South Australian Health & Medical Research Institute, North Terrace, Adelaide, SA 5000 Australia; 4 School of Biological Sciences, University of Adelaide, Adelaide, SA 5005 Australia; 5 Department of Respiratory & Critical Care Medicine, The Affiliated Hospital of Qingdao University, Qingdao, 266555 China; 6 Department of Thoracic Surgery, Qilu Hospital (Qingdao), Cheeloo College of Medicine, Shandong University, Qingdao, 266035 China; 7 Open Studio for Drug Research on Marine Natural Products, Pilot National Laboratory for Marine Science and Technology (Qingdao), Qingdao, People’s Republic of China

**Keywords:** Non-small-cell lung cancer, Transcriptional regulatory elements

## Abstract

PD-L1 is abnormally regulated in many cancers and is critical for immune escape. Fully understanding the regulation of PD-L1 expression is vital for improving the clinical efficacy of relevant anticancer agents. TGF-β plays an important role in the low reactivity of PD-1/PD-L1 antibody immunotherapy. However, it is not very clear whether and how TGF-β affects PD-L1 expression. In the present study, we show that TGF-β upregulates the expression of the transcriptional coactivator MRTF-A in non-small-cell lung cancer cells, which subsequently interacts with NF-κB/p65 rather than SRF to facilitate the binding of NF-κB/p65 to the *PDL1* promoter, thereby activating the transcription and expression of PD-L1. This leads to the immune escape of NSCLC cells. This process is dependent on the activation of the TGF-β signaling pathway. In vivo, inhibition of MRTF-A effectively suppresses the growth of lung tumor syngrafts with enrichment of NK and T cells in tumor tissue. Our study defines a new signaling pathway that regulates the transcription and expression of PD-L1 upon TGF-β treatment, which may have a significant impact on research into the application of immunotherapy in treating lung cancer.

## Lung cancer: A target to improve immunotherapy

Better understanding how a critical protein to allow cancer cells to escape immune system may aid in development of improved immunotherapies for lung cancer. The membrane protein PD-L1, expressed on tumor cells, helps them to evade the immune surveillance; existing treatments that block PD-L1 have very low efficacy for some patient partly due to re-expression of PD-L1. Jing Li at Ocean University of China in Qingdao and co-workers found that TGF-β up-regulated in tumor microenvironment boosts PD-L1 transcription and expression in an unusual way, namely, via MRTF-A-NF-κB/p65 axis. Blocking MRTF-A in a mouse model remarkably increased levels of immune cells targeting the tumor and slowed lung tumor growth. These results illuminate the mechanism of immune escape in lung cancers upon TGF-β, which may contribute to develop new treatment to synergize PD-L1 antibody therapy.

## Introduction

Programmed cell death protein 1 (PD-L1) is the dominant inhibitory ligand of PD-1, can be detected on hematopoietic cells, including macrophages, dendritic cells (DCs) and stromal cells, and is upregulated on the surface of tumor cells^1,2^. Its expression is regulated at the levels of gene transcription, mRNA translation, and protein stability^2–4^. The PD-L1/PD-1 axis has critical importance for immune escape in cancer development and in cancers with poor prognosis^5–7^. Blockade of PD-1 or PD-L1 with monoclonal antibodies (mAbs) can restore T and NK cell function and thereby reverse many of these phenomena^8,9^. PD-1/PD-L1 antibodies have gained clinical approval for the treatment of melanoma, non-small-cell lung cancer (NSCLC), renal cell carcinoma (RCC), Hodgkin’s lymphoma, urothelial carcinoma, and gastric cancer^10^. However, even in these tumor types, only a fraction of patients show objective clinical responses (e.g., to the anti-PD-1 monoclonal antibody nivolumab; complete response 0.7%, partial response 19.3%), among whom 15–35% will develop drug resistance^11^. Tumor cells can supplement or renew inactivated PD-L1 on their cell surface after antibody drug treatment^12,13^. In addition to immune escape, tumor-intrinsic PD-L1 can promote tumor cell proliferation, metastasis, and tumor stem cell formation^14,15^. Given that the expression of PD-L1 on tumor cells has predictive value for the response to antibody-based monotherapies in many studies, especially those on melanoma and NSCLC^6,16,17^, and because inhibition of PD-L1 expression in tumor cells in combination with anti-PD-L1 or anti-PD-1 antibody immunotherapy can effectively suppress the growth of various tumors^18,19^, fully understanding the regulation of PD-L1 expression is vital for improving the clinical efficacy of relevant anticancer agents.

Transforming growth factor-β (TGF-β) belongs to a superfamily of cytokines that are highly expressed in a variety of tumors. It can induce epithelial-mesenchymal transition (EMT) and stimulate tumor cell proliferation and survival^20,21^. Furthermore, TGF-β is central to immune suppression within the tumor microenvironment and plays a critical role in tumor immune evasion^22^. A large number of studies have shown that the low efficacy of PD-1 or PD-L1 antibody immunotherapy is closely related to the high expression of TGF-β in the tumor microenvironment^23,24^. There have been few reports about the relationship of TGF-β and PD-L1^25,26^. However, how TGF-β induces PD-L1 expression and in turn affects tumor immunity is still not clear.

Myocardin-related transcription factor-A (MRTF-A, also named *MKL1*) is a member of the myocardin family. MRTF-A is expressed in a wide range of tissues and has been reported to be a transcriptional coactivator of serum-response factor (SRF), thereby promoting the binding of SRF to the conserved cis regulatory element CC (A/T) 6GG (known as CarG box) of target genes, thus regulating their transcription^27–30^ and playing important roles in the growth and development of the organism^28^. MRTF-A can also promote tumor metastasis. Under basal conditions, MRTF-A binds to G-actin, preventing it from translocating to the nucleus^31^. Activation of the RhoA and TGF-β signaling pathways can induce the translocation of MRTF-A to the nucleus^32^, where it promotes the expression of EMT-related molecules and thus enhances the adhesion, migration, and invasion of tumor cells^32–35^. However, it is still unknown whether MRTF-A is a key coordinator in tumor immune responses.

Considering that PD-L1, TGF-β, and MRTF-A exhibit extensive cross-talk in the process of EMT, we set out to characterize whether and how TGF-β impacts PD-L1 expression and whether MRTF-A is involved in TGF-β/PD-L1-induced immune escape. In the present study, we demonstrate that TGF-β upregulates the expression of MRTF-A, which facilitates the binding of NF-κB/p65 rather than SRF to the PD-L1 promoter, thereby activating the transcription and expression of PD-L1 and allowing for the immune escape of NSCLC cells in vitro and in vivo. The process is dependent on activation of the noncanonical TGF-β pathway.

## Materials and methods

### Cell culture and reagents

A549, H1975, H1299, H446, HCT-8, HepG2, U87, MDA-MB-231, NK-92, and Lewis cell lines were purchased from the Shanghai Cell Bank. A549 cells were cultured in F-12K medium. H1975, H1299, H446, HCT-8, HepG2, and Lewis cells were cultured in RPMI 1640 medium. U87 cells were cultured in high-glucose DMEM. All cell lines were maintained in their respective medium with 10% fetal bovine serum except MDA-MB-231 cells (Leibovitz’s L-15 medium with 15% bovine calf serum) and NK-92 cells (minimum essential medium alpha (α-MEM) containing heat-inactivated 12.5% horse serum (Solarbio, China), 12.5% bovine calf serum, and 200 U/ml recombinant human IL-2 (PeproTech, USA)). All cell lines were grown to confluence at 37 °C in a humidified atmosphere with 5% CO_2_. Recombinant human TGF-β1 was purchased from PeproTech. Y27632, DORA, AKTi, U0126, and SB431245 were purchased from MedChemExpress. A cyclopeptide PD-1/PD-L1 inhibitor 3 (S8158) was purchased from Selleck^4,36^. Cell lysis buffer, ethylenediaminetetraacetic acid (EDTA), PMSF, MG132, a BCA Protein Assay Kit, cycloheximide, and IgG antibody were purchased from the Beyotime Institute of Biotechnology (Shanghai, China). Protein A/G conjugated to agarose was purchased from Santa Cruz Biotechnology. High-glucose DMEM, Leibovitz’s L-15, F12K medium, and α-MEM were obtained from Gibco (Rockville, MD, USA). Fetal bovine serum (FBS) was obtained from Gibco-Invitrogen (Grand Island, NY, USA). Rabbit and mouse mAb IgG XP® isotype controls and antibodies to detect β-catenin, p65, p-p65, Smad2, and Smad3 were obtained from Cell Signaling Technology. Anti-MRTF-A antibody was obtained from Proteintech. Anti-PD-L1 antibody was obtained from Novus Biologicals. Anti-CD8 antibody and anti-PD-L1 antibody were obtained from Abcam. Anti-NK1.1 antibody were obtained from Invitrogen. Anti-GAPDH antibody and the secondary antibodies were purchased from Hua Bio.

### Transfection with siRNA and DNA plasmids

siRNAs (si) directed toward MRTF-A, SRF, p65, β-catenin, Smad2, and Smad3 as well as scrambled negative control siRNA were purchased from GenePharma (Shanghai, China). Cells were seeded into six-well plates, incubated overnight, and then transiently transfected with siRNA or negative control using Lipofectamine 3000 (Invitrogen, Inc., Waltham, MA, USA) according to the manufacturer’s instructions. The sequences of the primers used in this study are listed in Table 1. The plasmid encoding MRTF-A (pcDNA3.1-MRTFA-myc/his) was described previously^30^.

**Table 1:** The sequences of primers used for real-time qPCR.

Genes	Sequence
si-MRTF-A	5′-GGACGACCTGTTTGACATT-3′
si-SRF	5′-CCCUGUUUCAGCAGUUCAGTT-3′
si-p65	5′- CAAG ATCAATGGCTACACA-3′
si-β-catenin	5′- GCTTGGAA TGAGACTGCTG-3′
si-Smad2	5′- GAAUUGAGCCACAG AGUAAUU-3′
si-Smad3	5′-GAGUUCACUCCACAUUCUCUU-3′
β-actin	F: 5′- CGAGATCCCTCCAAAATCAA-3′
	R: 5′-TTCACACCCATGACGAACAT-3′
MRTF-A	F: 5′-ACCGTG ACCAATAAGAATGC-3′
	R: 5′-CCGCTCTGAATGAGAATGTC-3′
SRF	F: 5′-TGCCTTCAGTAGGAACAAGC-3′
	R: 5′-ACTTCCATCTTG GCACCC-3′
PD-L1	F: 5′-AGACCACCACCACCAATTCC-3′
	R: 5′-TGGAGGATGTGCCAGAGGTA-3′
PD-L1 -Mut- luc	F: 5′-CGACGCGTGAGACA CTCTGAGAAACAGCAC-3′
	R: 5′-CCGCTCGAGCTCTGCCCAAGGCA GCAAATC-3′
Fragment I	F: 5′-CTCCATGCTCCTGCCAA-3′
	R: 5′-ACCTCAAGTGATCCGCC-3′
Fragment II	F: 5′-AAAAGGGAGCACACAGG-3′
	R: 5′-AAAAAGTCAGCAGCAGA-3′
Fragment III	F: 5′-ATATCAAGTTATGTCAA-3′
	R: 5′-GTCCCGCCCACCTCTGC-3′
Fragment IV	F: 5′-TGAGGTCAAGGAGTT-3′
	R: 5′-TGGGTTAGTGAATGG-3′
Fragment V	F: 5′-GGCTTTCTTAACCCTCAC-3′
	R: 5′-AACTTCATTTGCTTTGTC-3′
Fragment VI	F: 5′-GTCACAGAATCCACG-3′
	R: 5′-TAAAAAGTCAGCAGC-3′
SgRNA-MRTF-A	F: 5′-CACCGCTGACCAGCTCCGATCTCTC-3′
	R: 5′-AAACGAG AGATCGGAGCTGGTCAGC-3′

### Western blotting and coimmunoprecipitation (co-IP)

For the Western blotting analysis, cell lysates were prepared using 2× loading buffer on ice. Equal amounts of protein were separated through SDS-PAGE gels and then transferred to nitrocellulose (NC) membranes. The membranes were blotted with primary antibodies followed by secondary antibodies. Finally, the membranes were imaged by enhanced chemiluminescence.

For co-IP, the cells were lysed on ice for 30 min using Cell Lysis Buffer for Western blotting and IP with 1 mM PMSF. After centrifugation at 12,000 × *g* at 4 °C for 15 min, the supernatants were incubated overnight with the indicated antibody or IgG at 4 °C and then incubated for 2 h with protein A/G-agarose beads while rotating at 95 rpm. The beads were washed six times with washing buffer and resuspended in 2× loading buffer, and the pulled-down proteins were subjected to immunoblot analysis.

### Cell viability assay

Sulforhodamine B (SRB) assays were used to measure cell proliferation. For the SRB assay, adherent cells were seeded into 96-well plates (5000 cells/well). Cells were treated with TGF-β for the indicated times, and the SRB assay was used to evaluate cell mass and number. The absorbance at 515 nm was detected on a microplate reader (BioTek, Winooski, VT, USA).

### NK cell cytotoxicity assay

A549 and H1975 cells were cotreated with siNC or MRTF-A-specific siRNA in the presence or absence of TGF-β for 48 h. Then, the cells were reseeded, and after the cells had adhered to the plates, NK-92 cells were added at different effector-to-target ratios and incubated for 8 h at 37 °C. The lytic capacity of NK-92 cells was assessed by the release of lactate dehydrogenase (LDH) (Beyotime, Shanghai, China). Target cells were plated into 96-cell plates, and effector cells were added to the plates and incubated for 5 h. The ratios of effector cells to cancer cells (2.5:1, 5:1, and 10:1) were modified according to the requirements of each experiment. The cytotoxicity assay plate was centrifuged at 250 × *g* for 10 min. Then, 100 μl of supernatant was transferred to a well in a new 96-well plate, and 100 μl of substrate mix was added. After 30 min of incubation at room temperature, the absorbance at 490 nm was measured. A549 and H1975 cells that had been treated as described above were seeded into 48-well plates; effector cells were then added to the plates and incubated for 5 h. Afterward, NK-92 cells and cell debris were removed by washes with PBS, and live cancer cells were stained with crystal violet and photographed.

### Tissue samples

Human primary tumor specimens and matched adjacent normal mucosa samples were collected from seven lung cancer patients in Qilu Hospital of Shandong University under protocols (protocol KYLL-2018026) approved by the ethics committee. These specimens were surgically removed from patients, and the diagnosis was confirmed through pathological analysis. All pathological and clinical parameters were extracted from electronic medical records. Informed consent was obtained from all patients.

### Extraction of total RNA and RT-qPCR

Total RNA was extracted with TRIzol reagent (Invitrogen). cDNA was synthesized with M-MLV reverse transcriptase (Promega) and quantified by real-time qPCR using a Biosystems StepOne™ Real-Time PCR system and Fast SYBR Green Master Mix (Applied Biosystems), with β-actin as an internal control. PCR primers were designed with the NCBI online software Primer-BLAST and synthesized by TSINGKE. The PCR conditions were as follows: 94 °C for 2 min; 30 cycles at 94 °C for 30 s, 60 °C for 30 s and 72 °C for 1 min; and 72 °C for 10 min. The sequences of primers used in this study are listed in Table 1.

### Tissue microarray

A tissue microarray (TMA; HLugA150CS03) was purchased from Shanghai Outdo Biotech Co., Ltd. (Shanghai, China). This TMA comprises 150 cores from 75 patients with lung adenocarcinoma, including 75 tumor tissues and 75 corresponding adjacent tissues. The immunohistochemistry (IHC) score was calculated by multiplying the staining rate and intensity scores. PD-L1 ≤ 0.3 was classified as the low expression group, and PD-L1 > 0.3 was classified as the high expression group. MRTF-A ≤ 1.6 was classified as the low expression group, and MRTF-A > 1.6 was classified as the high expression group. Correlations were determined by Spearman’s coefficient.

### Luciferase reporter assay

pGL3-PD-L1-Luciferase reporter plasmids purchased from Addgene carry the wild-type PD-L1 promoter sequence (−2065 to −65) constructed with pGL3-basic firefly luciferase using Mlu1 and Xho1 sites. Additional luciferase reporter constructs of the PD-L1 promoter containing mutations in the putative binding site were generated using the QuikChange site-directed mutagenesis kit (TransGen). PD-L1-WT-luc was mutated from -GGGAAGTTCT- to -GGGAACAAGA-. The sequences of the primers used in this study are listed in Table 1. Cells were lysed, and luciferase activities were measured with a Luciferase Assay System (Promega) in a SynergyTM 4 luminometer (Bioteck).

### Analysis of cytokine levels by ELISA

A549 and H1975 cells were cotreated with siNC or MRTF-A-specific siRNAs and TGF-β for 48 h. Then, cells were seeded and allowed to adhere to the plates, and NK-92 cells were added at an effector-to-target ratio of 5:1 for 8 h at 37 °C. Perforin and granzyme B levels in the serum were measured using human perforin and granzyme B ELISA kits (Dakewe Biotech Co, Ltd. China), according to the manufacturer’s instructions.

### Chromatin immunoprecipitation (ChIP)

ChIP assays were carried out following a standard protocol^37^. Briefly, A549 cells were cross-linked with 1% formaldehyde (Sigma) for 15 min at room temperature with gentle shaking. The cells were harvested with a cell scraper, and chromatin was fragmented with a sonicator to an average length of 400 bp. Diluted chromatin fragments were incubated with anti-MRTF-A. After treatment with protein A beads (Santa Cruz Biotechnology), the beads were washed, and the bound material was eluted. The ChIP products were purified and measured by real-time qPCR. The sequences of the primers used in this study are listed in Table 1.

### Immunofluorescence

Cells were fixed with 4% paraformaldehyde for 30 min and then permeabilized with 0.3% Triton X-100 for 15 min. The cells were initially incubated with MRTF-A and p65 antibodies followed by FITC-labeled or Cy3-labeled secondary antibodies, washed three times with PBS and then counterstained with DAPI (Thermo Fisher, USA). The cells were examined under a fluorescence microscope. p65 and MRTF-A colocalization was calculated as the number of cells in which p65 and MRTF-A proteins were detected in the nucleus divided by the total number of cells.

### Nuclear fractionation

After cells were subjected to the indicated treatments, lysates were prepared and fractioned into cytoplasmic and nuclear components using a nuclear extraction kit (BestBio) according to the manufacturer’s instructions.

### Flow cytometry

A549 cells were digested by 0.25% trypsin with EDTA, resuspended in 100 µL of FACS buffer (1% BSA in PBS), and then incubated for 1 h with anti-PD-L1 antibody. After undergoing washing, the cells were incubated for 30 min in the dark with an Alexa Fluor 488-conjugated secondary antibody. After further washing, the cells were run through a flow cytometer (MFLO XDP; Beckman Coulter, USA), and the data were analyzed with FlowJo 8.8.6 software.

### CRISPR/Cas9 mediated knockout of MRTF-A

The lenti-CRISPR plasmid was requested from Addgene. Protospacer sequences of CRISPR/Cas9 knockout MRTF-A cells were designed by CRISPR DESIGN (http://crispr.mit.edu/). After transient transfection of CRISPR/Cas9 into cells using Lipofectamine 3000, treatment with puromycin (4 μg/mL) (Life Technologies) was employed for selection, and the cells were then expanded in a regular culture medium. The sequences of the primers used in this study are listed in Table 1.

### In vivo tumorigenicity assay

Female C57BL/6J mice aged 6–8 weeks were purchased from Beijing Vital River Laboratory Animal Technology Co., Ltd. The mice were housed in a pathogen-free animal facility and randomly assigned to the control or experimental group. For each cell line, 2 × 10^6^ cells were resuspended in 200 μl of PBS and then injected subcutaneously into C57BL/6J mice. Tumor formation was monitored every day by measuring the largest and smallest diameters of the formed tumors. No tumor exceeded the maximum size (2500 mm^3^) as indicated by the animal welfare committee and corresponding regulations. At the end of the experiments, the mice were euthanized by cervical dislocation under carbon dioxide inhalation, and the wet weights of each tumor were determined. Animal care practices and all experiments were reviewed and approved by the Committee on the Ethics of Animal Experiments of Ocean University of China.

### Immunohistochemistry

The tumor tissues in mice were fixed with formalin for subsequent IHC analysis to detect protein expression. Antibodies against MRTF-A, PD-L1, CD8, and NK1.1 were used for conventional IHC. The incubation of primary antibodies was performed on 4-μm thick, formalin-fixed, paraffin-embedded (FFPE) tissues. The primary antibodies were applied to the slides and then incubated in a humidified chamber at 4 °C overnight. The next day, the slides were washed, stained with 3,3′-diaminobenzidine, and independently reviewed under a microscope by two pathologists. Scores were calculated based on the intensity and percentage of positively stained tumor cells in the whole tissue staining according to the Fromowitz standard. The staining intensity was scored as 0 (no staining), 1 (weak staining), 2 (moderate staining), and 3 (strong staining). The percentage of positive cells was divided into four levels: 1 (0–25% positive cells), 2 (26–50% positive cells), 3 (51–75% positive cells), and 4 (76–100% positive cells). The product of the intensity and percentage scores represented the final staining score. Moreover, at least five fields of ×200 magnification from each core were reviewed for quantification^38^.

### Statistical analysis

All statistical analyses were performed using SPSS 17.0 (SPSS, Chicago, USA) with one-way ANOVA. All data are presented as the means ± S.D. *p* < 0.05 was accepted as significant. A *p* value < 0.05 is indicated with *, and a value <0.01 is indicated with **.

## Results

### TGF-β elevates the expression of PD-L1 and promotes tumor immune escape

**Fig. 1:**
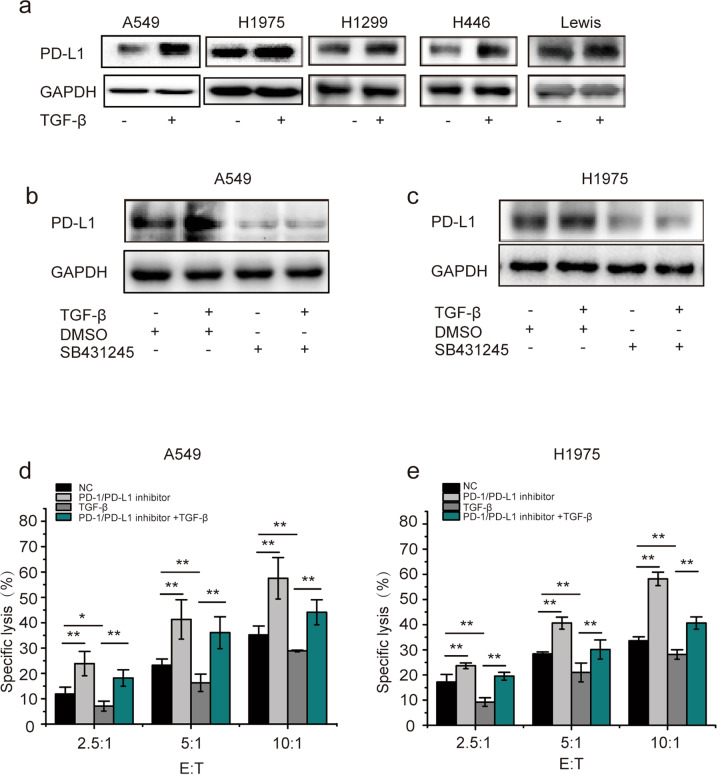
TGF-β elevates the expression of PD-L1 and promotes tumor cell immune escape. **a** The indicated lung cancer cells were treated with TGF-β (2.5 ng/ml), and PD-L1 expression was determined by Western blotting. **b** A549 and **c** H1975 cells were pretreated with vehicle (DMSO) or SB431245 (10 μM, 30-min preincubation) and then exposed to TGF-β for 8 h. PD-L1 expression was determined by Western blotting. **d** A549 and **e** H1975 cells were cotreated with PD-1/PD-L1 inhibitor and TGF-β for 8 h and then incubated with NK-92 cells for 8 h. Data are shown as the means ± S.D. using data from three independent experiments. *p < 0.05, **p < 0.01.

It has been reported that TGF-β plays an important role in immunosuppression and is associated with poor prognosis in cancer patients^24^. To investigate whether PD-L1 mediates TGF-β-induced immune evasion in NSCLC cells, we first examined the protein expression level of PD-L1. As shown in Fig. 1a, the expression of PD-L1 was increased in various lung cancer cell lines after TGF-β treatment. Furthermore, TGF-β increased PD-L1 expression in diverse types of tumor cells (Supplementary Fig. 1a). In A549 and H1975 cells, the TGF-β inhibitor SB431245 completely blocked the effect of TGF-β on PD-L1 expression, confirming that TGF-β is required for this effect (Fig. 1b, c). Kaplan–Meier analysis of the TCGA NSCLC database indicated that TGF-β and the expression level of PD-L1 were significantly negatively associated with patient overall survival (Supplementary Fig. 1b, c). In particular, there was a positive correlation between TGF-β and PD-L1 (Supplementary Fig. 1d). To ascertain the role of PD-L1 in immune evasion induced by TGF-β, the activity, and cytotoxicity of NK cells were determined in an in vitro assay in which NK-92 cells were cocultured with tumor cells with or without TGF-β treatment in the presence or absence of PD-1/PD-L1 inhibitor 3. The cell-killing ability of NK-92 cells was significantly decreased by TGF-β treatment at all effector:target (E:T) ratios compared with that of untreated controls for both A549 and H1975 cells, and this decrease was rescued by a PD-1/PD-L1 inhibitor. The PD-1/PD-L1 inhibitor alone was able to increase the cytotoxic activity of NK-92 cells, showing that the PD-1/PD-L1 axis contributes to immunosuppression between NK cells and cancer cells (Fig. 1d, e). Appropriate assays showed that TGF-β had no effect on the viability of A549 and H1975 cells (Supplementary Fig. 1e, f). All these results indicate that TGF-β promotes the expression of PD-L1, which contributes to the immune escape of tumor cells.

### The ubiquitin-proteasome system is not responsible for the TGF-β-induced increase in PD-L1 expression

**Fig. 2:**
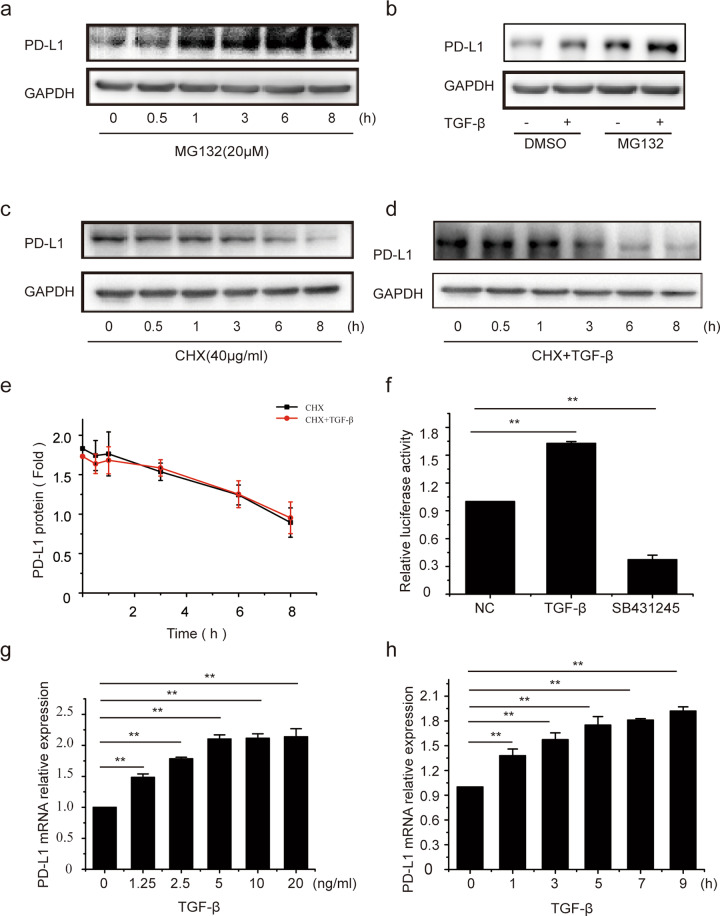
The ubiquitin-proteasome system is not responsible for the TGF-β-induced rise in PD-L1 expression. **a** A549 cells were treated with MG132 (20 μM) for the indicated times, and PD-L1 expression was analyzed by Western blotting. **b** A549 cells were treated with vehicle or MG132 and, where indicated, exposed to TGF-β for 8 h, and PD-L1 expression levels were assessed by Western blotting. **c** A549 cells were treated with CHX (40 μg/ml) for the indicated times, and PD-L1 expression was determined by Western blotting. **d** A549 cells were treated with vehicle or CHX in the presence of TGF-β for the indicated times, and PD-L1 expression was assessed by Western blotting. **e** Quantification of PD-L1 expression of (**c** and **d**) is shown. **f** A549 cells were transfected with luciferase promoter plasmid. After 24 h, they were then incubated in the presence or absence of TGF-β or SB431542 for 8 h and lysed, and luciferase activity was measured. **g** A549 cells were treated with the indicated concentrations of TGF-β, and then *PDL1* mRNA levels were determined by RT-PCR. **h** A549 cells were treated with TGF-β (2.5 ng/ml) for the indicated times, and *PDL1* mRNA levels were then determined by RT-PCR. Data are shown as the means ± S.D. using data from three independent experiments. *p < 0.05, **p < 0.01.

Recently, it has been reported that the ubiquitin-proteasome system regulates PD-L1 stability, which requires GSK3β-mediated phosphorylation of PD-L1 at Thr180 and Ser184^39^. As GSK-3β can be inhibited by TGF-β^40^, we suspected that TGF-β elevated the protein level of PD-L1 by suppressing its degradation. We therefore first tested the impact of proteasome inhibition on PD-L1 expression over different time periods. Immunoblots showed that the proteasome inhibitor MG132 caused a strong increase in PD-L1 protein levels in A549 cells (Fig. 2a). Similarly, TGF-β clearly induced the accumulation of the PD-L1 protein, and the effect of TGF-β combined with MG132 was significantly stronger than that of MG132 alone (Fig. 2b), which implies a potential role of TGF-β in inhibiting the proteolysis of PD-L1. Therefore, the half-life of the PD-L1 protein was measured in the absence or presence of TGF-β following cycloheximide (CHX) treatment to block the synthesis of new PD-L1. Immunoblots showed that the rate of decreased PD-L1 levels was very similar in the absence and presence of TGF-β (Fig. 2c, d), and no significant difference was observed between the two groups by quantitative analysis (Fig. 2e), clearly demonstrating that the TGF-β-induced rise in PD-L1 expression was not due to the altered degradation of the PD-L1 protein. To further explore how TGF-β regulates PD-L1 expression, A549 cells were transfected with the reporter plasmid PD-L1-WT-Luc, in which the transcription of luciferase was driven by the PD-L1 promoter. The results showed that TGF-β substantially increased luciferase activity (by ~1.7-fold), and the promoter activity of *PDL1* was dramatically decreased upon SB-431245 treatment (Fig. 2f). Therefore, we next examined the level of *PDL1* mRNA by qRT-PCR. As expected, the relative level of *PDL1* mRNA was elevated in a time- and concentration-dependent manner after TGF-β treatment (Fig. 2g, h). We conclude from these results that TGF-β activates the transcription of the *PDL1* gene.

### TGF-β promotes the transcription and expression of PD-L1 in an MRTF-A-dependent manner

**Fig. 3:**
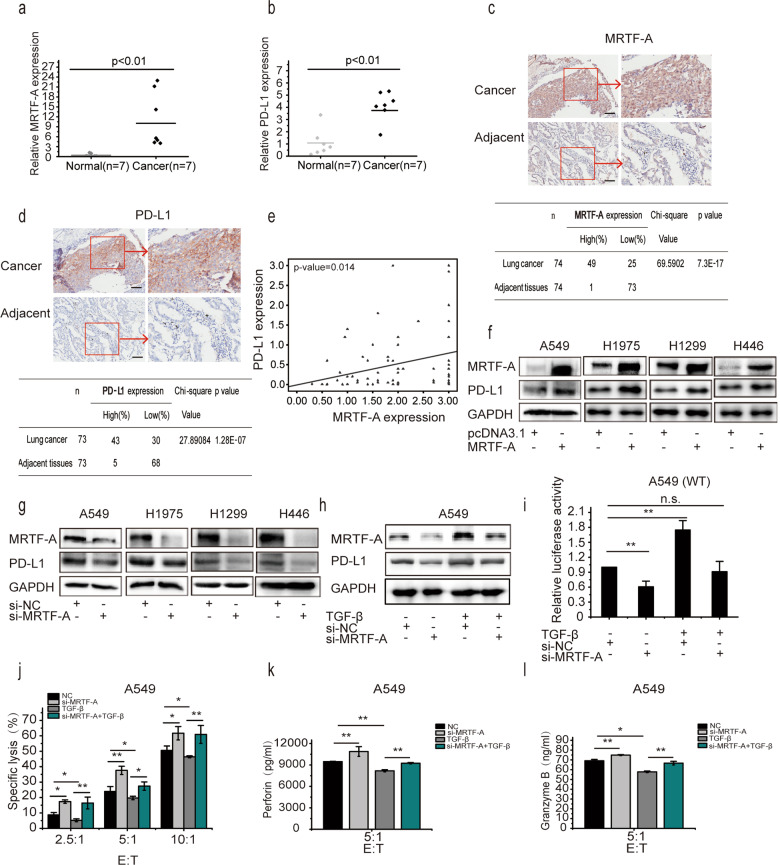
TGF-β promotes the transcription and expression of PD-L1 in an MRTF-A-dependent manner. **a**, **b** qRT-PCR was performed to detect the expression of MRTF-A (**a**) and PD-L1 (**b**) in 7 pairs of NSCLC tissues and adjacent tissues. **c**, **d** Representative images of MRTF-A (**c**) and PD-L1 (**d**) Immunohistochemical staining of 74 NSCLC specimens. The table shows the statistics of the differential expression of PD-L1 and MRTF-A. **e** Correlation between MRTF-A and PD-L1 protein levels in NSCLC patients was analyzed. **f** Cells were treated with pcDNA3.1 or MRTF-A plasmid. **g** Cells were treated with vehicle (si-NC) or MRTF-A siRNA. **h** A549 cells were pretreated with vehicle (si-NC) or MRTF-A siRNAs for 48 h and, where indicated, then exposed to TGF-β for 8 h, and changes in PD-L1 and MRTF-A expression were determined by Western blotting. **i** A549 cells were cotransfected with luciferase reporter plasmid along with si-NC or MRTF-A siRNA after 48 h and then incubated in the presence or absence of TGF-β for 8 h, and luciferase activity was measured. **j** A549 cells were cotreated with siNC or MRTF-A-specific siRNA with or without TGF-β and then incubated with NK-92 cells for 8 h. **k**, **l** Cells were treated as described in (**j**) except that the effector/target (E:T) ratio was 5:1, and cell-free culture supernatants of cells were then harvested to measure perforin and granzyme B by ELISA. Data are shown as the means ± S.D. using data from three independent experiments. *p < 0.05, **p < 0.01.

PD-L1, TGF-β, and MRTF-A engage in extensive cross-talk in the occurrence of EMT^26,41,42^, and MRTF-A, as a transcriptional coactivator, is employed to regulate the transcription and translation of multiple genes. Therefore, we aimed to characterize whether MRTF-A participates in TGF-β-promoted transcription and expression of PD-L1. TCGA database analysis showed that there were positive correlations between the levels of TGF-β and MRTF-A and between MRTF-A and PD-L1 (Supplementary Fig. 2a, b). Consistent with findings for TGF-β and PD-L1, statistical data clearly suggest that high MRTF-A expression was also significantly negatively associated with overall survival (Supplementary Fig. 2c). These results indicate that there were mutual associations between TGF-β, MRTF-A, and PD-L1. To assess the correlation between MRTF-A and PD-L1 in NSCLC patients, qRT-PCR analysis was performed in 7 pairs of NSCLC samples and adjacent mucosa. The data showed that the mRNA levels of MRTF-A and PD-L1 were much higher in the cancer samples (Fig. 3a, b). Furthermore, the expression of MRTF-A and PD-L1 in 75 NSCLC patients and normal control tissue samples was measured using a tissue microarray assay. Consistently, the protein levels of MRTF-A and PD-L1 were significantly higher in NSCLC tissue samples than in control normal tissue samples (Fig. 3c, d), and there was a positive correlation between MRTF-A and PD-L1 in NSCLC patients (Fig. 3e). Taken together, these results are consistent with the notion that MRTF-A is involved in the transcription of the *PDL1* gene and thus regulates the expression of PD-L1.

To study this further, a plasmid encoding MRTF-A was transfected into A549 and H1975 cells. In these MRTF-A-overexpressing cells, PD-L1 protein expression was clearly increased (Fig. 3f). Endogenous PD-L1 expression was significantly decreased in cells with MRTF-A knockdown by siRNAs (Fig. 3g), suggesting that MRTF-A plays an important role in the regulation of PD-L1 expression. Furthermore, upon TGF-β treatment, MRTF-A protein levels increased along with those of PD-L1. Importantly, TGF-β failed to promote PD-L1 expression when MRTF-A was depleted (Fig. 3h and Supplementary Fig. 2d), indicating that MRTF-A is required for TGF-β-mediated induction of PD-L1 expression in both A549 and H1975 cells. Then, we further examined whether the transcription of the PD-L1 gene was regulated by MRTF-A. Using the luciferase reporter system, TGF-β was found to readily increase PD-L1 promoter activity, which was abolished by siRNA-mediated downregulation of MRTF-A (Fig. 3i). The basal activity of the PD-L1 promoter state was also reduced after knockdown of MRTF-A; this is probably due to inhibition of the effect of TGF-β present in the culture medium (Fig. 3i). These findings suggest that MRTF-A promotes TGF-β-induced transcription of the PD-L1 gene. The role of MRTF-A in immune surveillance was again assessed in vitro in NK-92 cells cocultured with MRTF-A-depleted cancer cells. The cancer cell-killing ability of NK-92 cells after TGF-β treatment was significantly attenuated at all E:T ratios compared with that of control cells (Fig. 3j and Supplementary Fig. 2e–g), and MRTF-A siRNAs clearly counteracted the TGF-β-induced reduction in the killing ability of NK-92 cells. Perforin and granzyme B are released upon activation of NK cells^43^. Consistently, TGF-β treatment resulted in a significant reduction in perforin and granzyme B release, and treatment of cells with MRTF-A siRNAs clearly antagonized these effects (Fig. 3k. l, Supplementary Fig. 2h, i). Taken together, these results indicate that MRTF-A plays an important role in the transcription and thus the expression of PD-L1 induced by TGF-β and therefore helps to mediate immune escape.

### p65 is indispensable for TGF-β induced gene transcription of PD-L1

**Fig. 4:**
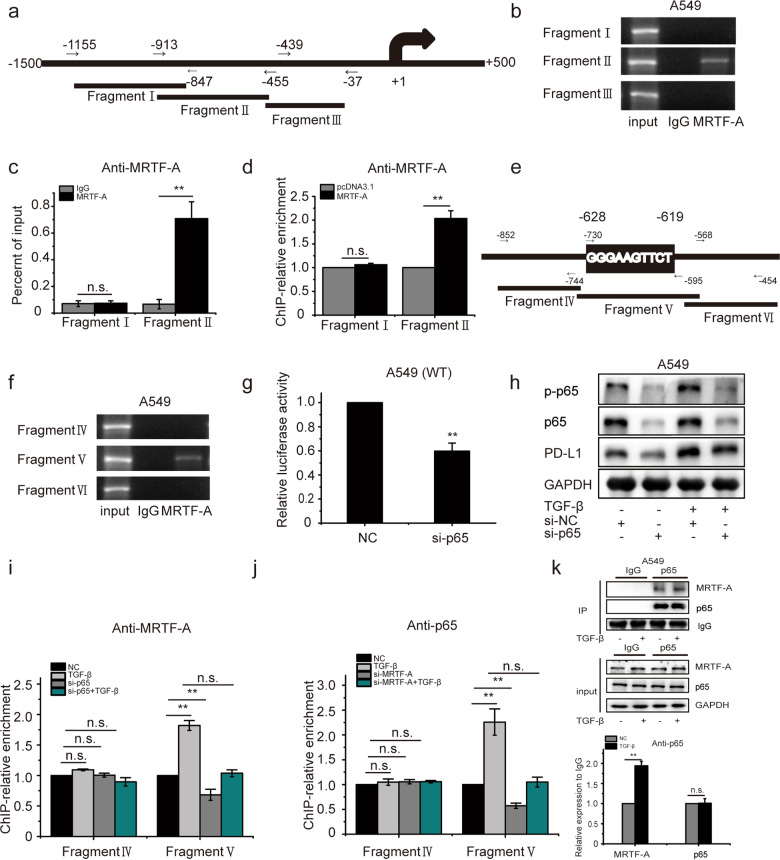
p65 is indispensable for TGF-β induced gene transcription of PD-L1. **a** Scheme depicting ChIP-PCR fragments of the PD-L1 promoter. **b** Results of the ChIP assays analyzed by PCR and electrophoresis or **c** by qPCR. Antibodies used in ChIP assays included those targeting MRTF-A. **d** A549 cells were treated with pcDNA3.1 or MRTF-A plasmid for 48 h. ChIP was performed with antibodies against MRTF-A. **e** Schematic of the PD-L1 promoter with the locations of three ChIP-PCR fragments depicted. **f** Results of the ChIP assays as analyzed by PCR and electrophoresis. **g** A549 cells were cotransfected with luciferase promoter plasmid and si-p65; after 48 h, luciferase activity was measured. **h** A549 cells were pretreated with vehicle (si-NC) or p65 siRNAs for 48 h and, where indicated, then exposed to TGF-β for 8 h, and protein levels were determined by Western blotting. **i** A549 cells were transfected with p65 siRNAs, followed by treatment with TGF-β. ChIP assays were performed with the MRTF-A antibody. **j** A549 cells were transfected with MRTF-A siRNAs, followed by treatment with TGF-β. ChIP assays were performed with the p65 antibody. **k** Cells were incubated in the presence or absence of TGF-β, and cell lysates were prepared and immunoprecipitated using anti-p65 antibodies. Data are shown as the means ± S.D., using data from three independent experiments. *p < 0.05, **p < 0.01.

MRTF-A usually forms a complex with SRF to facilitate the binding of SRF to CArG boxes on DNA, thereby driving the expression of hundreds of target genes^29,30,44^. To examine the involvement of SRF in the transcription of the PD-L1 gene, SRF was knocked down in A549 cells. As shown in Supplementary Fig. 3a, b, depletion of SRF had no effect on PD-L1 mRNA or PD-L1 protein levels, suggesting that the MRTF-A-SRF complex is not essential for activating the transcription and translation of PD-L1. We, therefore, speculate that MRTF-A requires other transcription factor(s) to regulate the transcription of the PD-L1 gene. To clarify this point, a series of ChIP assays were performed. The position of the ChIP-qPCR fragments covering the PD-L1 promoter (−1500 bp to the transcription start site) is depicted in Fig. 4a. Compared with the corresponding IgG isotype, MRTF-A antibodies efficiently coprecipitated fragment II of the PD-L1 promoter but not fragments I and III (Fig. 4b, c). To ensure that fragment II accounted for TGF-β-induced PD-L1 transcription and expression, MRTF-A was overexpressed, and ChIP assays were performed as described before. In cells overexpressing MRTF-A, the enrichment of PD-L1 promoter fragment II was clearly increased (Fig. 4d). In addition, the pull-down of fragment II by MRTF-A antibody was enhanced at higher concentrations of TGF-β (Supplementary Fig. 3c). Taken together, these results indicate that MRTF-A physically interacts with the PD-L1 promoter in the region of fragment II and that this interaction is enhanced by TGF-β.

To accurately analyze the characteristics of fragment II, we performed ChIP assays with three domains covering the entirety of fragment II. The position of the ChIP-qPCR domains is shown in Fig. 4e. This experiment revealed that fragment V was bound by MRTF-A, indicating that MRTF-A interacts with the PD-L1 promoter at fragment V (Fig. 4f). After inspecting the sequence of fragment V, we found that it contains a *cis*-acting element of p65 (GGGAAGTTCT). In addition, based on the use of the luciferase reporter system for PD-L1, transcriptional activity of the PD-L1 promoter was impaired by depleting p65 by siRNA (Fig. 4g). These results suggest that p65 may participate in the transcription and expression of PD-L1. To further assess whether p65 mediates the transactivation of the PD-L1 promoter upon TGF-β treatment, we constructed luciferase reporter vectors with a mutant p65 *cis*-acting element in the PD-L1 promoter and transfected them into A549 cells. The data show that TGF-β treatment alone was unable to induce transcription of the luciferase reporter from the mutant version of the *PDL1* promoter and that SB431245, si-MRTF-A, and si-NF-кB were ineffective in blocking transcription from the mutant version of the *PDL1* promoter compared to the control promoter in A549 cells (Supplementary Fig. 3d). Furthermore, p65 depletion significantly impaired TGF-β-stimulated expression of PD-L1 in A549 and H1975 cells (Fig. 4h and Supplementary Fig. 3e). All these results support the notion that p65 plays a critical role in regulating the transcription and expression of PD-L1 in response to TGF-β. To confirm that an interaction exists between MRTF-A and p65 in cells, we knocked down p65 in A549 cells and then examined the enrichment of the PD-L1 promoter by ChIP with an antibody against MRTF-A. This revealed that the enrichment of fragment V after TGF-β treatment was nearly completely blocked (Fig. 4i). Similarly, the enrichment of fragment V was also nearly completely inhibited in MRTF-A-depleted cells, as detected by ChIP with an antibody against p65 (Fig. 4j). Furthermore, at the protein level, there was strong basal binding between p65 and MRTF-A, which was further substantially elevated in the presence of TGF-β, as detected by coimmunoprecipitation (Fig. 4k). All these results indicate that the MRTF-A-p65 complex can form and is promoted by TGF-β, and p65 functions as an executive transcription factor with MRTF-A in the induction of transcription of the PD-L1 gene by TGF-β.

### TGF-β-induced PD-L1 expression requires noncanonical pathways

**Fig. 5:**
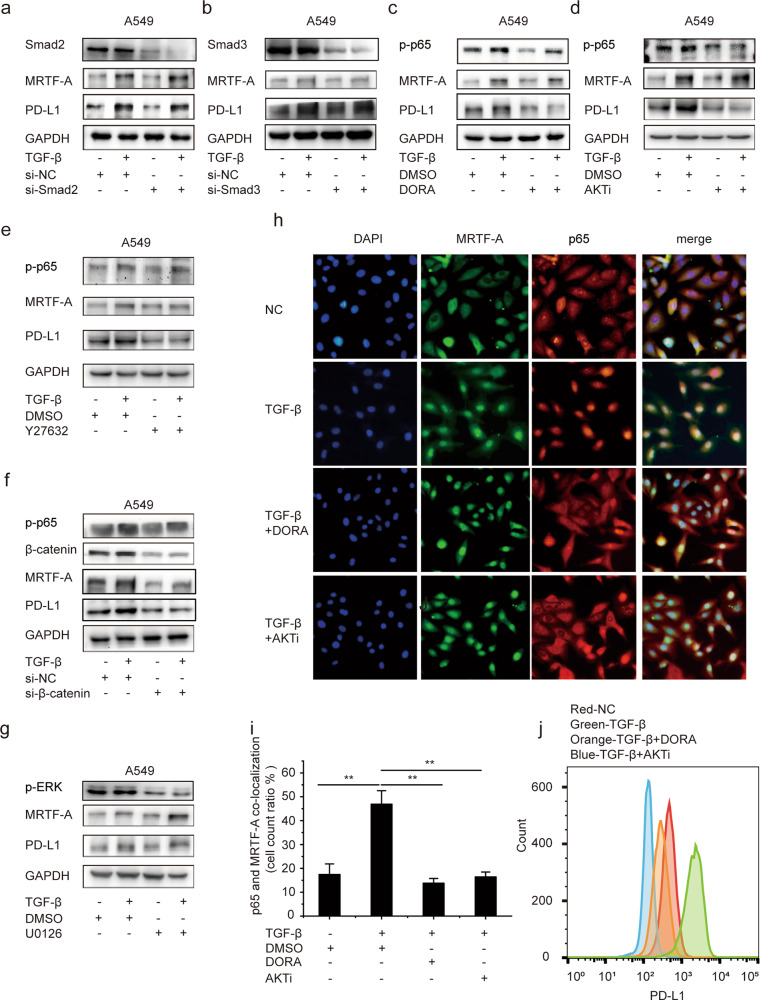
TGF-β-induced PD-L1 expression requires noncanonical pathways. A549 cells were transfected with si-Smad2 (**a**), si-Smad3 (**b**) or si-β-catenin (**f**) for 48 h and then exposed to TGF-β for 8 h, and changes were determined by Western blotting. **c**–**g** A549 cells were pretreated with DORA (**c**), AKTi (**d**), Y27632 (**e**), or U0126 (**g**) for 30 min and, where indicated, exposed to TGF-β for 8 h, and changes were determined by Western blotting. **h** A549 cells were pretreated with vehicle (DMSO), AKTi, or DORA where indicated and exposed to TGF-β for 8 h. Cells were then stained for endogenous MRTF-A, p65, or nuclei (using the marker DAPI), and the localization of MRTF-A and p65 was observed by immunofluorescence microscopy. **i** The graph shows the quantification of (**h**). **j** A549 cells were treated as described in (**h**), and PD-L1 expression was analyzed by flow cytometry. Data are shown as the mean ± SD, using data from three independent experiments. *p < 0.05, **p < 0.01.

TGF-β activates Smad2/3-dependent (canonical) and Smad2/3-independent (noncanonical) signaling pathways^45^. To assess the role of the canonical signaling pathway in TGF-β-induced PD-L1 expression, Smad2 was depleted with specific siRNAs in A549 and H1975 cells. PD-L1 protein levels were not significantly altered following Smad2 knockdown (Fig. 5a and Supplementary Fig. 4a). Similarly, Smad3 siRNA reduced Smad3 expression but failed to reduce the expression of PD-L1 (Fig. 5b and Supplementary Fig. 4b). To further clarify the role of the canonical signaling pathway in TGF-β-induced PD-L1 expression, we used a luciferase gene reporter for PD-L1 and found that siRNA against Smad2 or Smad3 did not affect the TGF-β-induced increase in luciferase activity (Supplementary Fig. 4c, d). These results suggest that TGF-β-induced PD-L1 expression involves noncanonical signaling pathways of TGF-β.

Noncanonical pathways include RhoA/ROCK1, PI3K/Akt, Wnt/β-catenin, MAPK/p38, or MAPK ERK1/2, all of which mediate different effects of TGF-β on tumor cells^35,45,46^. Thus, we assessed the TGF-β-induced change in PD-L1 expression in the absence or presence of various inhibitors, including the selective p38 MAPK inhibitor doramapimod (DORA), the Akt inhibitor AKTi-1/2 (AKTi), the MEK inhibitor U0126, the Rho kinase inhibitor Y-27632 and β-catenin siRNA. In A549 and H1975 cells, we observed an upregulation in PD-L1 expression upon TGF-β treatment, and DORA, AKTi, Y-27632 and β-catenin siRNAs significantly suppressed TGF-β-induced PD-L1 expression (Fig. 5c–f and Supplementary Fig. 4e–h). In contrast, U0126 did not exert any significant effect either alone or in the presence of TGF-β (Fig. 5g and Supplementary Fig. 4i). It has been reported that β-catenin and Rho can regulate the expression and nuclear localization of MRTF-A, respectively^35,47^. AKT and p38 provoke the phosphorylation and consequent activation of p65^48,49^. In agreement with these reports, we noted that β-catenin siRNA decreased the expression of MRTF-A and PD-L1 without changing p-p65 levels. Y27632 decreased the expression of PD-L1 without any change in total MRTF-A expression or the level of p-p65, and DORA and AKTi inhibited the activation of p65 synchronizing with a decrease in PD-L1 without alteration of MRTF-A. These results indicate that signaling via Akt, p38 MAP kinase, Rho, and Wnt-β-catenin significantly contributes to MRTF-A expression, nuclear translocation, and p65 activation in the TGF-β-induced expression of PD-L1.

p65 is primarily localized to the cytoplasm in an inactive state and translocates into nuclei upon activation^49^. Immunoblots showed that TGF-β caused a strong increase in p-p65 protein levels in A549 cells (Supplementary Fig. 4j), and there was a remarkable increase in the nuclear p65 level upon TGF-β treatment. (Supplementary Fig. 4k). Thus, we further observed the nuclear localization of endogenous MRTF-A and p65 after TGF-β treatment in A549 cells using an immunofluorescence assay. TGF-β triggered the rapid and marked accumulation of MRTF-A and p65 in the nucleus with increased colocalization of the two proteins, while DORA and AKTi clearly inhibited the translocation of p65 into the nucleus, indicating that these inhibitors impeded their colocalization following treatment with TGF-β (Fig. 5h, quantified in 5i). In line with the immunofluorescence results, PD-L1 expression was enhanced after TGF-β treatment, and this was largely prevented by DORA or AKTi (as detected by FACS; Fig. 5j). These findings indicate that noncanonical pathways of TGF-β signaling involving Akt, p38 MAP kinase, Rho, and Wnt-β-catenin synergistically increase nuclear MRTF-A and p65 levels, contributing to the induction of PD-L1 expression by TGF-β.

### Knockdown of MRTF-A inhibits tumor immune escape and tumorigenesis in vivo

**Fig. 6:**
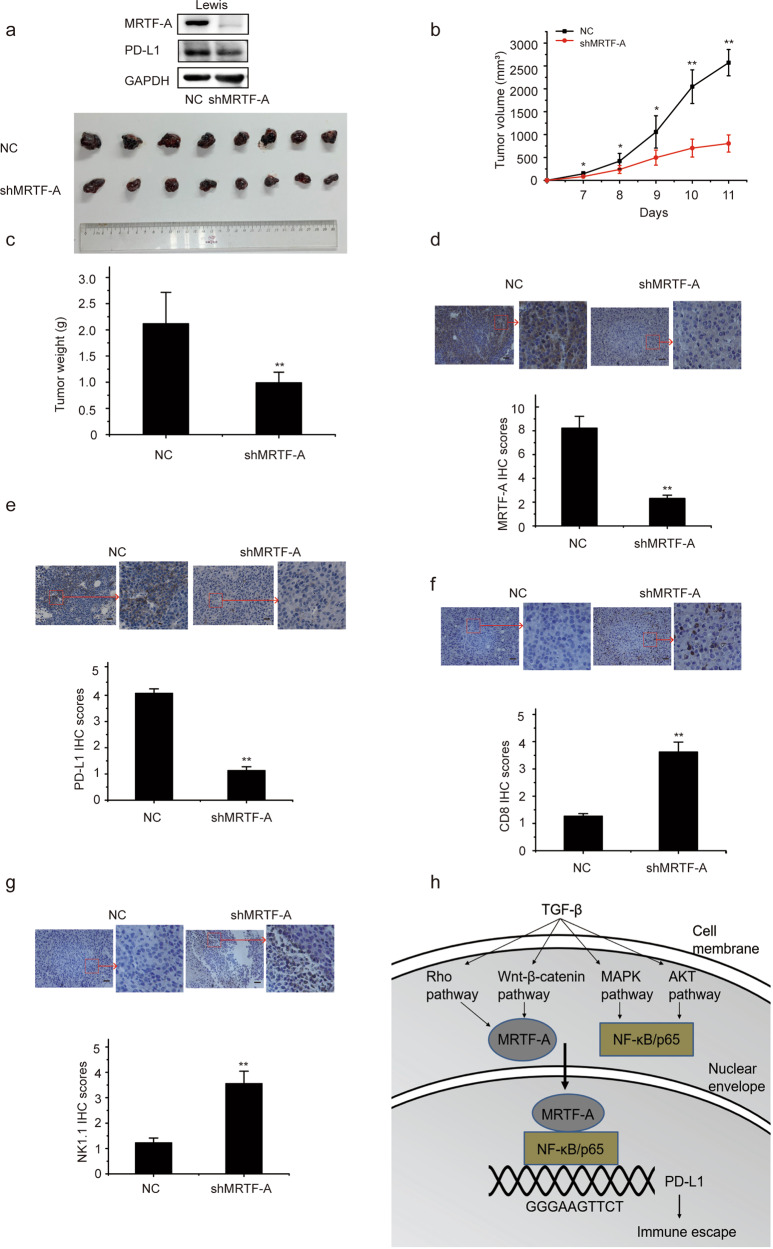
Knockdown of MRTF-A inhibits tumor immune escape and tumorigenesis in vivo. **a** PD-L1 and MRTF-A expression was determined by Western blotting. Photographs of tumors derived from NC and MRTF-A knockdown cells in mice. *n* = 8 mice per group. The experiment was repeated three times. **b** The tumor volume was calculated. **c** Weights of tumors. **d**, **e** MRTF-A and PD-L1 expression was examined in syngrafts containing si-NC and MRTF-A knockdown cells. **f**, **g** CD8 and NK1.1 expression was examined in syngrafts with si-NC or MRTF-A knockdown cells. **h** A schematic working model based on our findings. Data are shown as the means ± SD using data from three independent experiments. *p < 0.05, **p < 0.01.

To address the role of MRTF-A in mediating the upregulation of PD-L1 expression that contributes to immune escape and NSCLC tumorigenesis in vivo, we established Lewis cell lines with stable MRTF-A knockdown (sh-MRTF-A) and negative control cells (NC) using the CRISPR-Cas9 genome-editing system to study the biological functions of MRTF-A in a murine model. We initiated tumor growth by subcutaneously injecting 1 × 10^6^ sh-MRTF-A cells or NC cells into C57BL/6J mice. The tumors formed by Lewis cells with sh-MRTF-A grew significantly more slowly than those formed by control cells, as indicated by the smaller tumor volumes and weights (Fig. 6a–c). Immunohistochemical staining was performed to determine the expression of MRTF-A and PD-L1 in mouse tumor tissues. As expected, the expression of MRTF-A and PD-L1 clearly decreased in the syngraft tumors with MRTF-A knockdown (Fig. 6d, e). We also examined the enrichment of NK and T cells in the tumor tissue. The amounts of T cells (assessed as CD8-positive) in the tumor tissue significantly increased in these syngraft tumors after depletion of MRTF-A (Fig. 6f). The expression of the NK cell marker molecule NK1.1 was significantly increased in the syngraft tumors with MRTF-A knockdown compared with tumors in the control group (Fig. 6g). These results suggest that more NK and T cells are recruited into tumor tissues when MRTF-A is knocked down, supporting the in vitro findings that MRTF-A promotes the expression of PD-L1 and plays an important role in the immune escape of NSCLC cells.

## Discussion

The tumor-intrinsic expression of PD-L1 is aberrantly regulated in many cancers, and the underlying mechanisms include genomic alterations, for example, gene copy number amplification, dysregulated transcription, and 3′-UTR disruption;^2^ constitutive oncogenic signaling activation (e.g., the loss of PTEN expression); and consequent activation of the PI3K/AKT pathway, activation of the RAS/MAPK pathway, inhibition of p53 signaling, and upregulation of OCT4^2,50^. Other mechanisms include extrinsic factors, such as increases in TGF-β, IFN-γ and TNF-α^3^, and epigenetic mechanisms, such as aberrant DNA methylation and histone modifications^2,51^. Our studies provide a novel mechanism for the regulation of PD-L1 transcription and PD-L1 expression upon TGF-β treatment. We demonstrate that in the presence of TGF-β, MRTF-A acts as a coactivator of the transcription factor p65 to associate with the promoter of the PD-L1 gene and thereby promote the transcription and expression of PD-L1 (Fig. 6h).

TGF-β activates downstream signals through canonical and noncanonical pathways^45^. In the classical pathway, TGF-β first forms a complex with TβRII/TβRI, then phosphorylates Smad2 or Smad3 and finally translocates to the nucleus to promote the transcription of related genes. Noncanonical pathways include RhoA/ROCK1, PI3K/Akt, Wnt/β-catenin, MAPK/p38 or MAPK ERK1/2, which mediate different effects of TGF-β on tumor cells^35,45,46^. Several pathways (not including MEK/ERK1/2) together contribute to the activation of the transcription and expression of PD-L1. The nuclear translocation of MRTF-A upon activation of Rho-ROCK signaling has been reported^52,53^. We previously defined the regulation of MRTF-A expression by Wnt/β-catenin signaling^35^. In this report, we further verified that TGF-β-induced activation of PI3K/AKT and p38 MAPK triggers the NF-κB signaling pathway by increasing the phosphorylation and promoting the nuclear translocation of p65. Following activation of these signaling pathways by TGF-β, MRTF-A translocates into the nucleus, where it binds to intranuclear p65, facilitating the association of p65 with the promoter of the PD-L1 gene to activate the transcription and expression of PD-L1.

Transcriptional signaling networks are orchestrated and fine-tuned through multiple interactions of transcription factors with subsets of cofactors, thereby assembling multiprotein complexes to negatively or positively control transcriptional output. The function of the MRTF coactivators of the transcription factor SRF has attracted much attention^44,54^. However, siRNA-mediated depletion of SRF did not reduce the transcription or expression of PD-L1 in our studies. In addition to SRF, MRTFs bind to some members of the Smad family of transcription factors to exert specific cellular functions^55^. For example, Smad3 binds the B-box of MRTF-A and MRTF-B and redirects its activity to a newly identified *cis*-element in the slug gene promoter during TGF-β-induced EMT^41,55^. In our study, Smad2 or Smad3 were not involved in TGF-β-induced PD-L1 transcription. Furthermore, they did not affect the expression of MRTF-A or the level of phosphorylated p65. These results indicate that other transcription factors are required by MRTF-A to mediate the upregulation of PD-L1. Using ChIP with an MRTF-A antibody and biochemical experiments, we identified that the p65 subunit of NF-κB is a transcription factor that combines with MRTF-A to activate the transcription of PD-L1. It has been reported that p65 binds the B/Q region of myocardin to prevent the formation of a myocardin/SRF complex on DNA, thereby repressing myocardin-mediated activation of genes in myocardial and smooth muscle cells^56^. In view of this, we have uncovered a novel positive function of the interaction between p65 and MRTF-A.

NK cells play important roles in innate immune responses toward tumors^57^. In the tumor microenvironment, NK cells display higher expression of PD-1, and PD-1/PD-L1 blockade might therefore reverse the dysfunctional status of NK cells in this context, adding to the benefits of enhanced T cell responses upon PD-1/PD-L1 blockade^9^. In this article, we documented that MRTF-A plays a critical role in governing PD-L1 expression to regulate NK and T cell-mediated immune surveillance in vitro and in vivo, thereby effectively suppressing the growth of lung tumor syngrafts under depletion of MRTF-A.

In summary, our data show for the first time the role of MRTF-A as a key orchestrator in regulating TGF-β-induced PD-L1 transcription in NSCLC cells and reveal that p65 is a new executive transcription factor for MRTF-A in coactivating PD-L1 transcription. Furthermore, our data identify that the multiple noncanonical pathways downstream of TGF-β cooperatively induce the expression, activation, nuclear translocation, and interaction of MRTF-A and its interaction with p65, thereby promoting the transcription and expression of PD-L1 and ultimately assisting the immune escape of certain cancer cells. Our findings are likely to have a significant impact on research on tumors in immunotherapy, since targeting the MRTF-A/p65 axis may be a promising strategy to enhance the efficacy of checkpoint immunotherapy against lung cancer and other types of cancers^42^.

## Supplementary information

Supplementary Information
