# Supplementary material for: MRTF-A-NF-κB/p65 axis-mediated PDL1 transcription and expression contributes to immune evasion of non-small-cell lung cancer via TGF-β
Source: Exp Mol Med. 2021 Sep 21;53(9):1366–78. doi: 10.1038/s12276-021-00670-3 (PMC8492728; doi:10.1038/s12276-021-00670-3)
Supplement: Supplementary file 1 — Supplementary Information [file 12276_2021_670_MOESM1_ESM.docx]

**
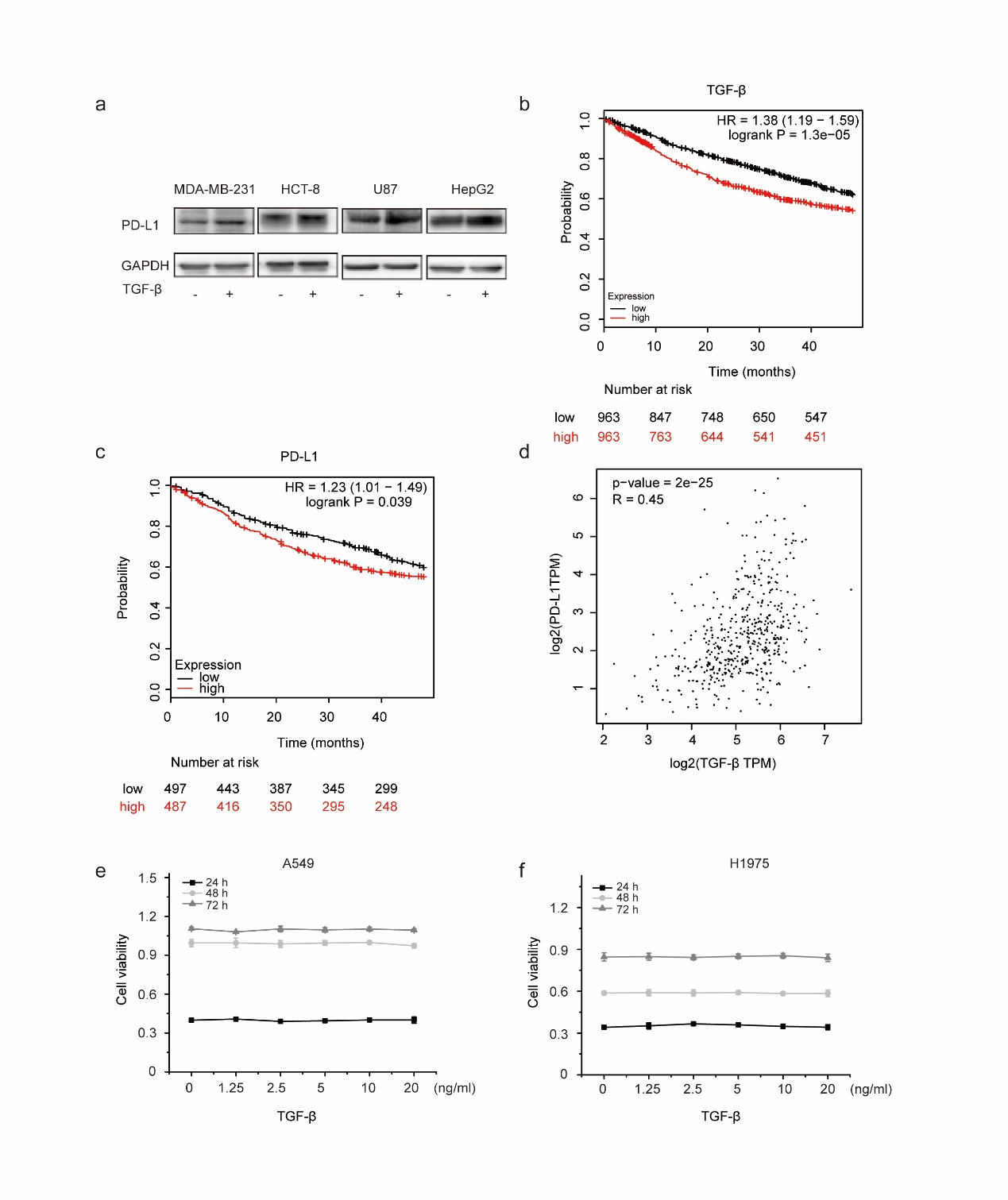
**

**Supplementary Figure. 1.** (a) Different types of tumor cells were treated with TGF-β and PD-L1 expression was determined by Western blotting. (b) Kaplan–Meier analysis of overall survival of NSCLC patients in TCGA database with high versus low TGF-β expression. (c) Kaplan–Meier analysis of overall survival of NSCLC patients in TCGA database with high versus low PD-L1 expression. (d) Correlation between TGF-β and PD-L1 mRNA levels in NSCLC patients was analyzed using the TCGA database. (e, f) A549 cells (e) and H1975 cells (f) were treated with the indicated concentrations of TGF-β for different times. The effect of cell viability was analyzed by SRB assay. Data represent three independent experiments.

**
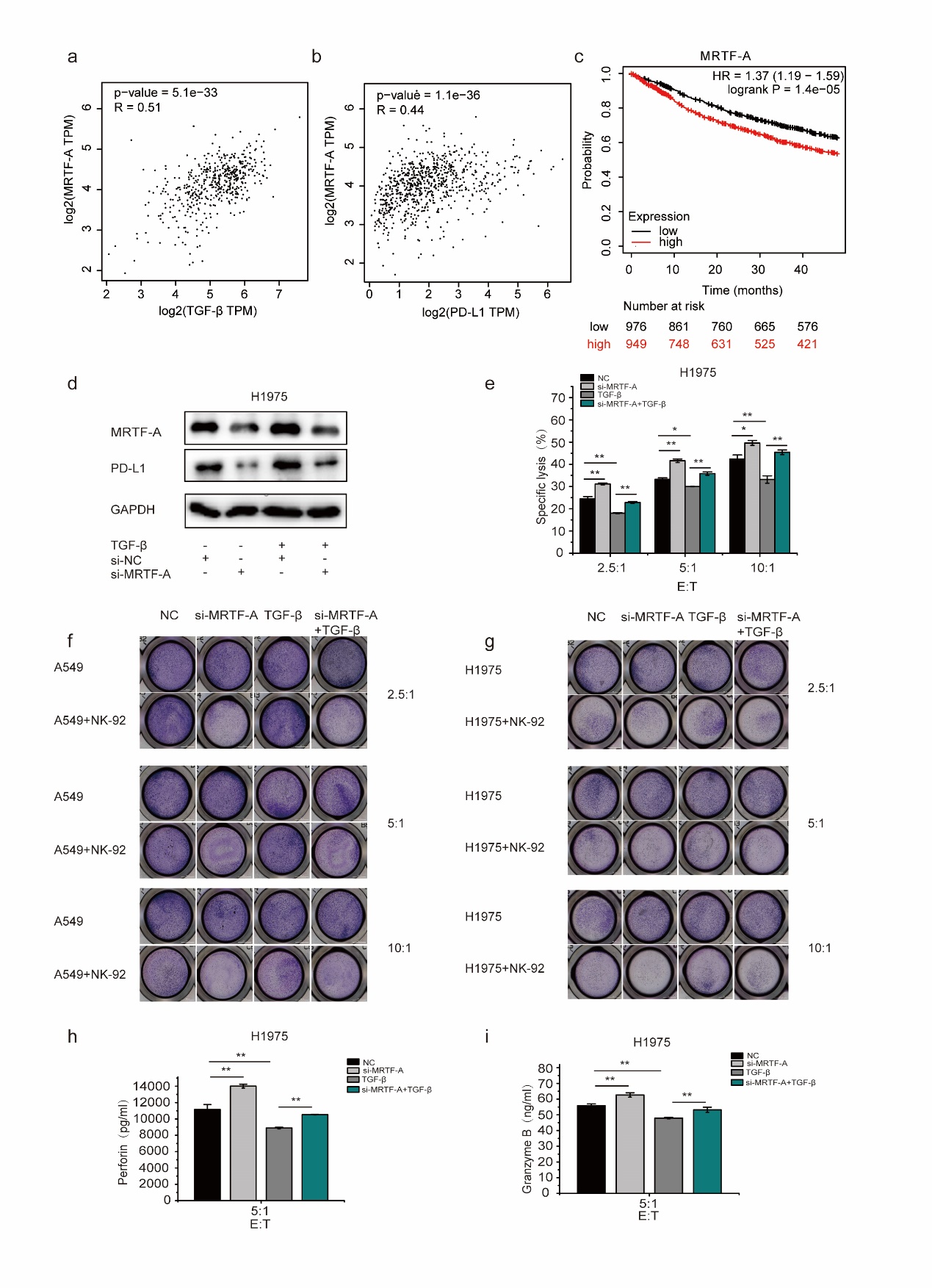
**

**Supplementary Figure. 2.** (a) Correlation between TGF-β and MRTF-A mRNA levels in NSCLC patients was analyzed using information from the TCGA database. (b) Correlation between MRTF-A and PD-L1 mRNA levels in NSCLC patients was analyzed using data from TCGA. (c) Kaplan–Meier analysis of overall survival of NSCLC patients in TCGA database with high versus low MRTF-A expression. (d) H1975 cells were pretreated with vehicle (si-NC) or MRTF-A siRNAs; after 48 h, where indicated, cells were exposed to TGF-β for 8 h, and changes in PD-L1 and MRTF-A expression were then determined by Western blotting. (e) H1975 cells were co-treated with siNC or MRTF-A-specific siRNA with or without TGF-β, and then seeded at 5 × 10^3^ cells per well in a 96-well plate and incubated with NK-92 cells for 8 h at various effector/target (E:T) cell ratios as indicated. (f and g) A549 and H1975 cells transfected with siNC or MRTF-A-specific siRNA were cultured in the presence or absence of TGF-β for 8 h, and then seeded at 3 × 10^4^ cells per well in a 48-well plate and incubated with NK-92 cells for 8 h at various effector/target (E:T) cell ratios as indicated, before being subjected to crystal violet staining. (h and i) Cells were treated as described in (d) except that the effector/target (E:T) ratio was 5:1, and then cell-free culture supernatants of cells were harvested to analyze perforin and granzyme B by ELISA. Data are shown as the mean ± S.D., using data from three independent experiments. *, p < 0.05, **, p < 0.01.

**
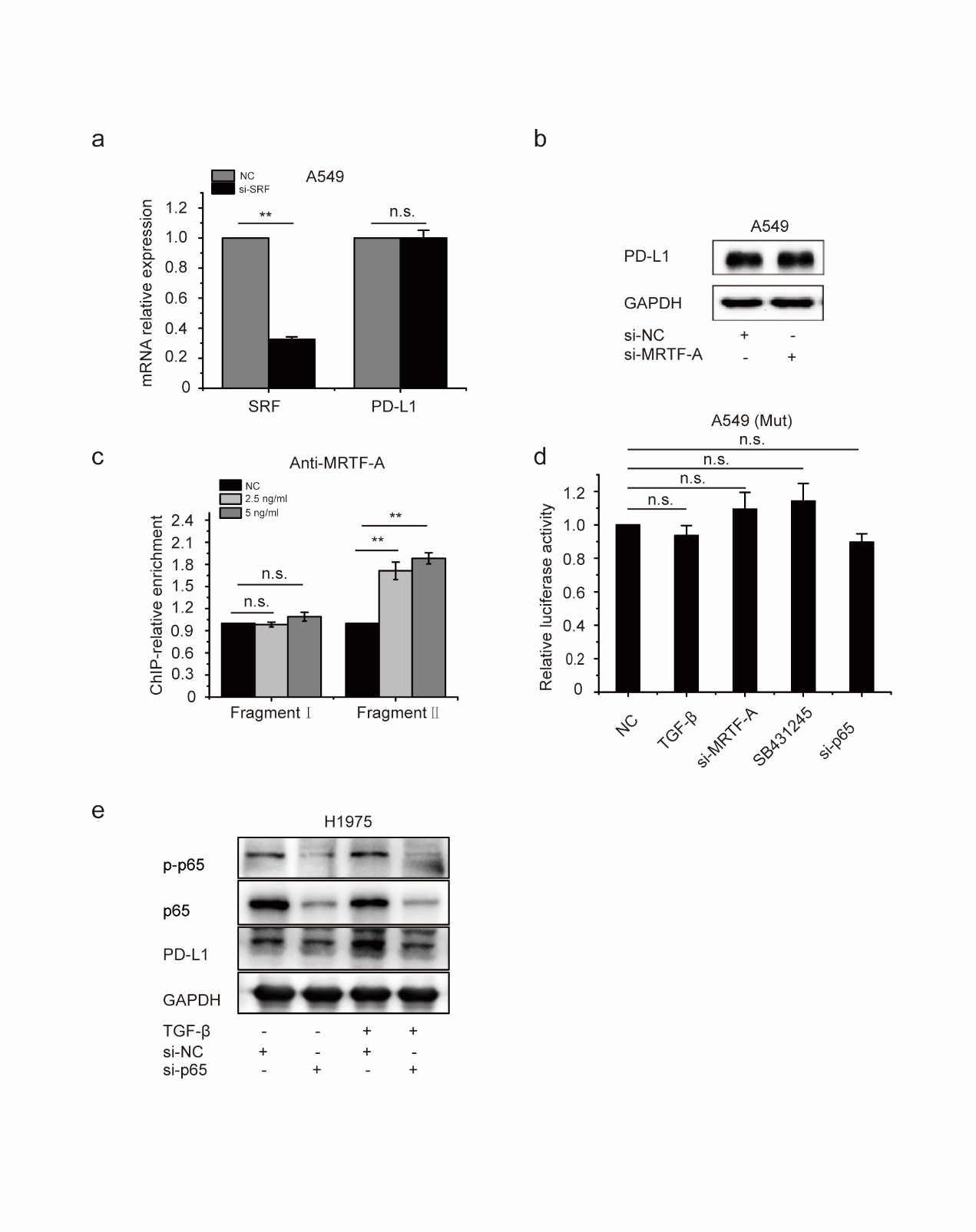
**

**Supplementary Figure. 3.** (a) A549 cells were transfected with si-SRF; after 48 h, SRF and PD-L1 mRNA levels were determined by RT-PCR. Data are normalized to β-actin as loading control. (b) A549 cells were transfected with SRF siRNAs; after48 h, PD-L1 expression was determined by Western blotting. (c) A549 cells were treated with different concentrations of TGF-β. ChIP assays were performed with the MRTF-A antibodies. (d) A549 cells were co-transfected with mutant luciferase reporter plasmid of PD-L1 along with si-MRTF-A or si-p65 for 48 h, and TGF-β or SB431245 for 8 h. Then luciferase activity was measured. (e) H1975 cells were pretreated with vehicle (si-NC) or p65 siRNAs; after 48 h, where indicated, cells were exposed to TGF-β for 8 h, and protein levels were determined by Western blotting. Data are shown as the mean ± S.D., used data from three independent experiments. *, p < 0.05, **, p < 0.01.

**
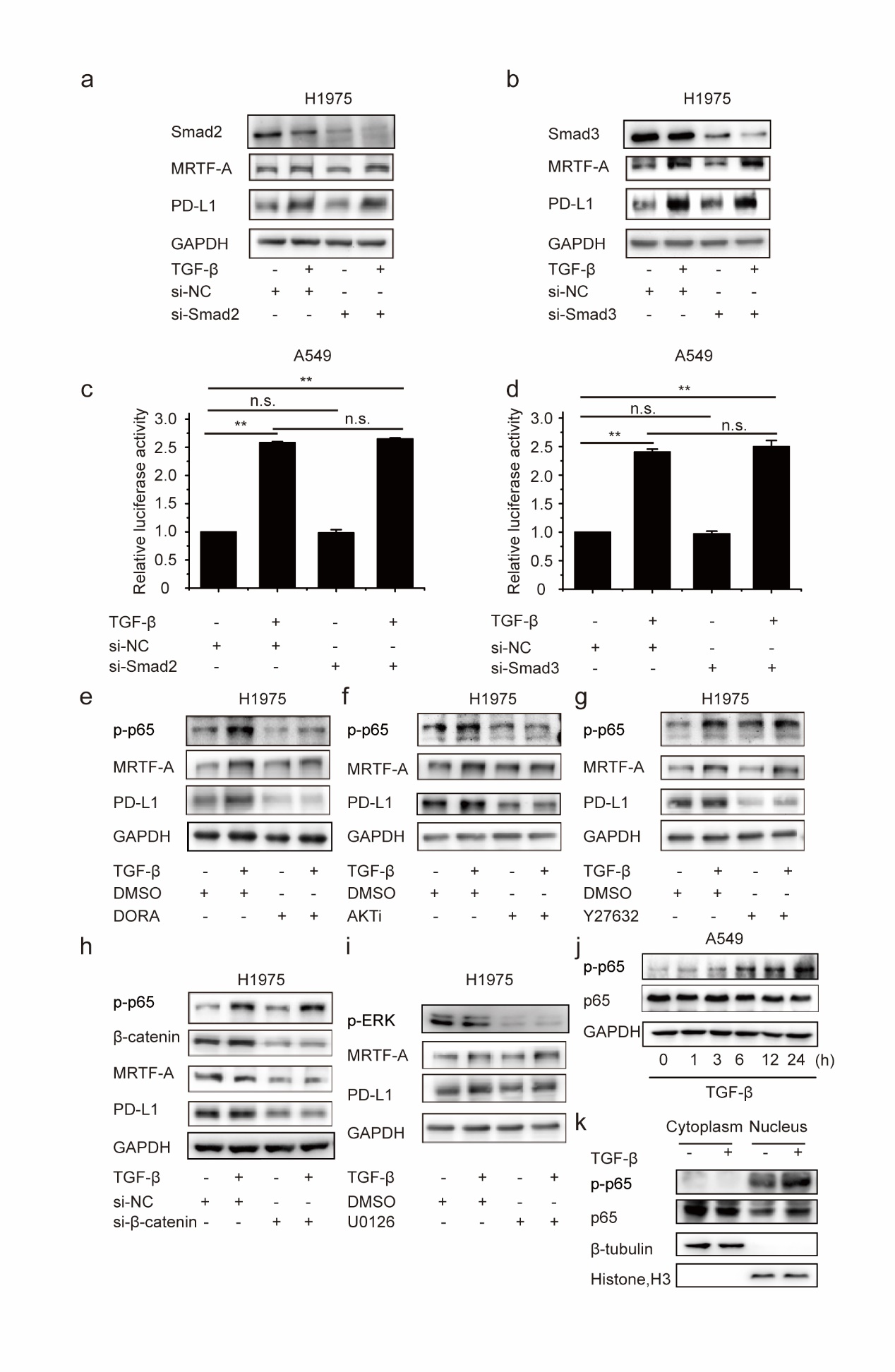
**

**Supplementary Figure. 4.** H1975 cells were transfected with si-Smad2(a), si-Smad3(b) or si-β-catenin (h) for 48 h and then, where indicated, exposed to TGF-β for 8 h, and changes were determined by Western blotting. Non-related (si-NC) was used as negative control. (c and d) A549 cells were transfected with luciferase promoter reporter vector and/or si-Smad2 (a) or si-Smad3 (b) for 48 h, then incubated in the presence or absence of TGF for 8 h, and luciferase activity was measured. (e-i) H1975 cells were pretreated with DORA (e), AKTi (f), Y27632 (g), U0126 (i) for 30 min and, where indicated, exposed to TGF-β for 8 h, and changes were determined by Western blotting. (j) A549 cells were treated with TGF-β for the indicated times, and p-p65 expression was analyzed by Western blotting. (k) Cells were treated with or without TGF-β, and cytoplasmic and nuclear fractions were separated and probed for p65, tubulin as a cytosolic marker, and histone H3 as a nuclear marker. Data are shown as the mean ± S.D., used data from three independent experiments. *, p < 0.05, **, p < 0.01.
